# Noradrenergic Signaling in Astrocytes Influences Mammalian Sleep Homeostasis

**DOI:** 10.3390/clockssleep4030028

**Published:** 2022-07-07

**Authors:** Ashley M. Ingiosi, Marcos G. Frank

**Affiliations:** 1 Department of Translational Medicine and Physiology, Elson S. Floyd College of Medicine, Washington State University, Spokane, WA 99202, USA; ashley.ingiosi@wsu.edu; 2 Gleason Institute for Neuroscience, Washington State University, Spokane, WA 99202, USA

**Keywords:** adrenergic receptor, astrocyte, homeostasis, noradrenaline, siesta, sleep, sleep deprivation

## Abstract

Astrocytes influence sleep expression and regulation, but the cellular signaling pathways involved in these processes are poorly defined. We proposed that astrocytes detect and integrate a neuronal signal that accumulates during wakefulness, thereby leading to increased sleep drive. Noradrenaline (NA) satisfies several criteria for a waking signal integrated by astrocytes. We therefore investigated the role of NA signaling in astrocytes in mammalian sleep. We conditionally knocked out (cKO) β2-adrenergic receptors (β2-AR) selectively in astrocytes in mice and recorded electroencephalographic and electromyographic activity under baseline conditions and in response to sleep deprivation (SDep). cKO of astroglial β2-ARs increased active phase siesta duration under baseline conditions and reduced homeostatic compensatory changes in sleep consolidation and non-rapid eye movement slow-wave activity (SWA) after SDep. Overall, astroglial NA β2-ARs influence mammalian sleep homeostasis in a manner consistent with our proposed model of neuronal–astroglial interactions.

## 1. Introduction

Sleep homeostasis refers to a regulatory process that controls the accumulation and discharge of sleep drive as a function of prior wake time [1]. In contrast to the circadian biological clock, which is anatomically and molecularly well-characterized, the biological substrates of the sleep homeostat are poorly understood [1]. Recent findings indicate that astrocytes are part of the mammalian sleep homeostat. For example, inhibition of astroglial gliotransmission reduces the accumulation of sleep need as measured by reductions in compensatory changes in sleep time and non-rapid eye movement sleep (NREMS) slow-wave activity (SWA; i.e., delta) in the electroencephalogram (EEG) [2]. Subsequent investigations showed that astrocytes dynamically change their activity across the sleep–wake cycle [3,4,5,6], and manipulation of this activity influences sleep homeostasis [3,7,8]. 

Less is known about the transduction pathways that link neural activity in wakefulness with changes in astrocytes necessary for sleep homeostasis. One possibility is that this reflects a feedback loop consisting of a neuronal waking signal and an astroglial transducer/integrator of this signal that results in the release of somnogens [8,9]. The waking signals are presently unknown, but a plausible scenario is that they satisfy at least three basic criteria. First, they should be released at higher rates in wakefulness vs. sleep, second, they should be linked to sleep homeostasis, and third, they should activate astrocytes *in vivo* (e.g., by elevating intracellular calcium (Ca^2+^)) [2]. The monoamine noradrenaline (NA) satisfies these criteria as NA brain concentrations peak during normal wakefulness (or after sleep deprivation (SDep)) and reach a nadir during sleep [10,11,12,13]. In addition, NA is a potent activator of astrocytes *in vivo* [14], and depletion of brain NA reduces the homeostatic response to SDep [15]. 

We therefore explored the role of astroglial NA beta-2 adrenergic receptors (β2-AR; *Adrb2*) in murine sleep expression and homeostasis using inducible Cre-lox technology (conditional knockout: cKO). The β2-AR receptor is expressed in mature astrocytes *in vivo* and, when activated, increases intracellular Ca^2+^ [16]. In GFAP-CreERT2 mice, an astrocyte-specific promoter (GFAP) drives Cre expression, and Cre activity is controlled by an engineered fusion protein that binds to a synthetic ligand (tamoxifen) [17]. When these mice are crossed with floxed lines (i.e., with a gene of interest flanked by loxP sites), the floxed gene is deleted only in astrocytes in the offspring after tamoxifen treatment (<1% recombination in neurons) [17]. We crossed GFAP-CreERT2 mice with Adrb2^flox^ mice to produce bigenic animals for astroglial cKO of β2-AR (and see [18]). Our principal finding is that cKO of astroglial β2-ARs reduced compensatory (homeostatic) responses to SDep. This is consistent with our proposed model of an astroglial sleep homeostat [8,9].

## 2. Results

### 2.1. Baseline Changes in Sleep Expression Following cKO of Astroglial β2-ARs

We assessed cKO of astroglial β2-ARs in two ways. First, we previously verified via immunofluorescence that Cre recombination in GFAP-Cre/ERT2^Tg+/−^ mice occurred selectively in astrocytes [3], which is in agreement with other previous work [19,20,21,22,23,24]. Second, we further verified β2-AR cKO with an *in vivo* functional astroglial Ca^2+^ imaging assay similar to what we used in our previous studies [3]. Because there are currently no reliable antibodies for β2-AR [25], we were not able to immunohistochemically directly quantify the reduction of β2-AR expression. Instead, we used the functional assay to determine that Ca^2+^ activity of frontal cortex astrocytes—which we previously showed changes with vigilance states and sleep need [3]—in cKO mice was impaired in response to the β-AR agonist isoproterenol (5 mg/kg; intraperitoneally; Ca^2+^ event frequency: U = 1350.00, *p* < 0.001; mean ΔF/F: U = 810.00, *p* < 0.001) (Figure S1).

**Figure 1:**
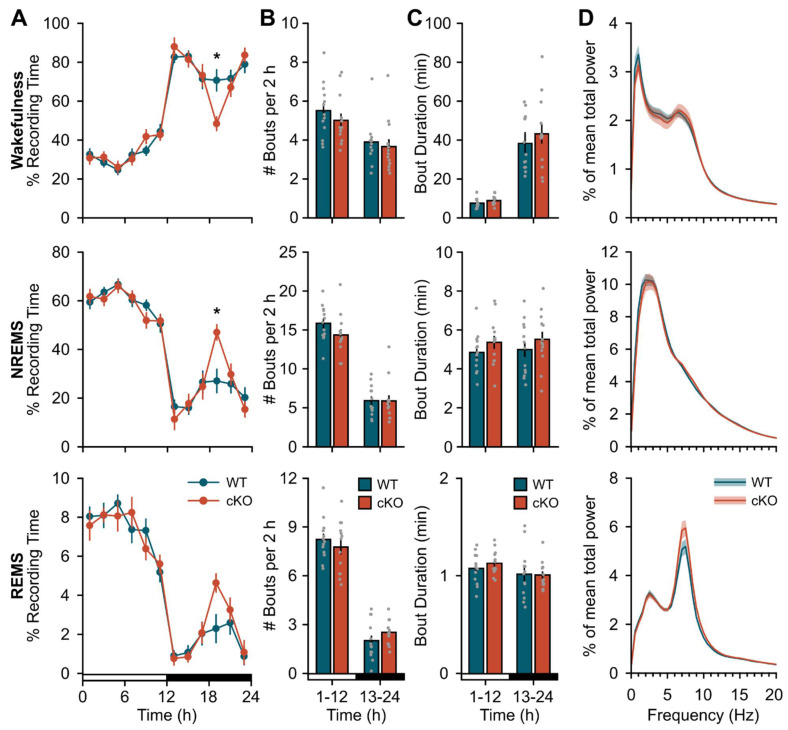
Conditional deletion of astroglial β2-ARs increases duration of dark period siesta. (**A**) Time spent in wakefulness (top), NREMS (middle), and REMS (bottom) expressed as a percentage of total recording time in 2 h bins. (**B**) Average bout frequency and (**C**) bout duration for wakefulness (top), NREMS (middle), and REMS (bottom) during the 12 h light and dark periods. (**D**) Normalized EEG spectral power for wakefulness (top), NREMS (middle), and REMS (bottom) over 24 h. Open and closed bars on the *x*-axis denote the light and dark periods, respectively. Values are means ± SE from *n* = 13 WT and *n* = 13 cKO mice. * *p* < 0.05 (repeated measures ANOVA).

We next found cKO of astroglial β2-ARs resulted in subtle changes in baseline sleep expression (Figure 1). The most prominent change was an unexpected increase in sleep time during the dark phase (Figure 1A; genotype x time effects, wake: F(3.77,90.54) = 2.84, *p* = 0.031; NREMS: F(5,120) = 2.86, *p* = 0.018; rapid eye movement sleep (REMS): F(3.14,75.45) = 1.911, *p* = 0.132). This occurred within a specific window corresponding to the ‘siesta’—a ~2 h sleep period that typically occurs during the latter half of the active phase—reported in mice of this C57Bl/6J background strain [26,27]. We also observed slight changes in REMS EEG activity in the theta (5–9 Hz) band, but this was limited to only one frequency (Figure 1D; genotype effect, F(1,24) = 4.43, *p* = 0.046; 9 Hz, *p* = 0.049). We did not find significant differences in 24 h patterns in baseline NREM SWA (Figure S2A). Measures of body mass (Figure S2C), diurnal/nocturnal patterns of core body temperature (Figure S2D), and core body temperature range (minimum, wild type (WT): 33.98 ± 0.05 °C, cKO: 34.06 ± 0.19 °C, t(5) = −0.23, *p* = 0.83; maximum, WT: 37.78 ± 0.31 °C, cKO: 37.86 ± 0.10 °C, t(5) = −0.37, *p* = 0.73) showed no gross abnormalities in the cKO mice.

### 2.2. Changes in Sleep Homeostasis Following cKO of Astroglial β2-ARs

**Figure 2:**
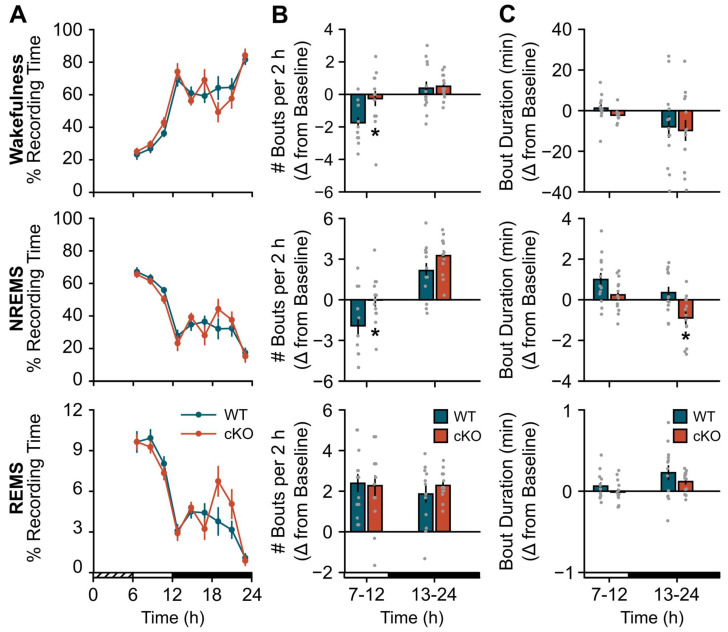
Conditional deletion of astroglial β2-ARs blunts the compensatory response to sleep deprivation (SDep): sleep consolidation. (**A**) Time spent in wakefulness (top), NREMS (middle), and REMS (bottom) after 6 h SDep expressed as a percentage of total recording time in 2 h bins. (**B**) Average bout frequency and (**C**) bout duration shown as change from baseline (SDep–baseline differences) during 6 h (light period) and 12 h (dark period) bins post-SDep recovery for wakefulness (top), NREMS (middle), and REMS (bottom). Cross-hatched bars on the *x*-axis denote the 6 h SDep period. Open and closed bars on the *x*-axis denote the recovery phase light and dark periods, respectively. Values are means ± SE from *n* = 13 WT and *n* = 13 cKO mice. * *p* < 0.05 (repeated measures ANOVA).

**Figure 3:**
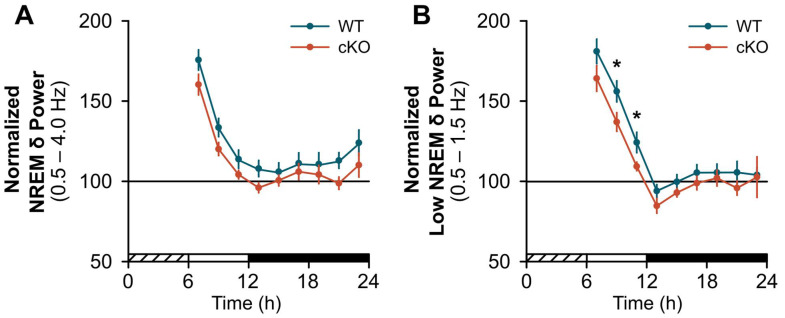
Conditional deletion of astroglial β2-ARs blunts the compensatory response to sleep deprivation (SDep): NREM slow-wave activity (SWA). Normalized NREM SWA for (**A**) delta (δ) bands (0.5–4.0 Hz) and (**B**) low delta bands (0.5–1.5 Hz) shown in 2 h bins post-SDep. Cross-hatched bars on the *x*-axis denote the 6 h SDep period. Open and closed bars on the *x*-axis denote the recovery phase light and dark periods, respectively. Values are means ± SE from *n* = 13 WT and *n* = 13 cKO mice. * *p* < 0.05 (repeated measures ANOVA).

cKO mice displayed a blunted response to a standard probe of sleep homeostasis (i.e., 6 h SDep) [28]. While compensatory changes in sleep time were similar between cKO and WT mice (Figure 2A), the normal homeostatic increase in sleep continuity (as measured by bout frequency and duration) after SDep did not occur in cKO mice. For example, cKO mice did not show an expected decrease in wakefulness or NREMS bout frequency during the light period (Figure 2B; wake: genotype x time effect, F(1,24) = 4.51, *p* = 0.044; NREMS: genotype effect, F(1,24) = 7.06, *p* = 0.014) nor an increase in NREM bout duration during the dark period (Figure 2C; NREMS: genotype effect, F(1,24) = 9.20, *p* = 0.006) compared to controls. Similarly, the normal homeostatic increase in NREM SWA was blunted in cKO mice in frequency bands previously shown to be impacted by astroglial manipulation (Figure 3, light period: genotype effect, F(1,24) = 4.37, *p* = 0.047) [2,3]. Further inspection of NREMS EEG spectra during SDep recovery did not show any difference above 4 Hz (Figure S2B).

## 3. Discussion

We investigated the role of NA signaling in astrocytes in sleep homeostasis using conditional and selective deletion (cKO) of astroglial β2-ARs in mice. We find several changes in cKO mice indicating that astroglial NA β2-AR signaling influences sleep expression and regulation. Our main findings are that cKO mice had a blunted response to SDep, similar to mutant mice with reductions in gliotransmission or astroglial intracellular Ca^2+^ [2,3]. This did not appear to be due to gross abnormalities in the mutant mice as they showed normal body mass and core temperature (relative to WT controls). We discuss aspects of these findings in more detail below.

### 3.1. Monoamines and Sleep Homeostasis

As reviewed in Greene and Frank [23], several neuromodulators have been proposed as mediators of sleep homeostasis (and/or NREM SWA). Serotonin (5HT), for example, has long been implicated in sleep drive [29], as its brain concentrations are highest in wakefulness and low in sleep, pharmacological blockade of 5HT2 receptors increases NREM SWA, and embryonic KO of the 5HT2c receptor increases the compensatory response to SDep [30]. On the other hand, it is not clear that 5HT is necessary and sufficient for sleep homeostasis because while brain depletion of 5HT (using p-chlorophenylalanine) combined with SDep reduces compensatory changes in sleep time [31], this has no effect on NREM SWA [32]. A relatively stronger case can be made for NA. NA brain concentrations are also maximal in waking, reach a nadir during sleep, and increase with SDep [10,11,12,13]. In rats, depletion of NA using the neurotoxin N-(2-chloroethyl)-N-ethyl-2-bromobenzylamine (DSP-4) reduces SDep-induced compensatory changes in NREM SWA and cortical immediate early gene (IEG) expression [15]. The latter further suggests a link between NA and sleep homeostasis because cortical IEG expression rises and falls with sleep drive with a time course comparable to NREM SWA [33]. However, the precise signaling pathways involved in this process are unclear (see [34] for discussion). Deletion of the dopamine β-hydroxylase (*Dbh*) gene in mice—which results in the complete absence of NA—reduces compensatory changes in sleep duration but has no effect on NREM SWA after SDep [34]. The contribution of different NA receptors has also been minimally explored with respect to sleep homeostasis and has chiefly been investigated with pharmacology which can lack specificity and is highly dependent on the site of action (see [34] for discussion). This complicates the role of NA in sleep homeostasis as different NA receptors can change wakefulness or sleep time via direct or indirect paths (e.g., changes in thermoregulation) [35] but may not provide insights about the sleep homeostat per se. Therefore, the current role of monoamines in sleep homeostasis is unresolved.

### 3.2. Astroglial Signaling and Sleep Homeostasis

One possible resolution for these disparate findings is that the effects of neuromodulators depend on their activity in neurons *and* glia. Astrocytes may represent a parallel level of brain organization that influences sleep expression and regulation [8]. Astrocytes express several subtypes of NA receptors *in vivo*, including α1-, α2-, β1-, β2-, and β3-ARs [36,37]. The activation of both α- and β-ARs trigger similar intracellular cascades in astrocytes, albeit through different pathways. Therefore, activation of either receptor subtype could provide a waking signal that increases sleep drive as recently proposed [8,9]. There are, however, several differences in signaling between astroglial α- and β-ARs that suggest that β-ARs may be the critical subtype in this process. Astroglial α1- and α2-ARs have greater affinity for NA [38] and trigger rapid changes in intracellular astroglial Ca^2+^ via activation of G_q_ and G_i_ pathways, respectively [39]. This makes them well-suited for detecting fast changes in NA release associated with specific behavioral contexts (e.g., during learning, attention) or modulating rapid changes in synaptic transmission. In contrast, the β2-AR has lower affinity for NA [38] and is coupled to G_s_ signaling which upregulates cyclic adenosine monophosphate (cAMP) [39], and secondarily Ca^2+^ [16], under slower time scales [38]. For example, astroglial cAMP takes longer to peak and decline compared to Ca^2+^ in response to optogenetic stimulation of NA cortical terminals or fear-conditioned foot shock *in vivo* [38]. Theoretically, this slower G_s_ signaling pathway would be more sensitive to cumulative changes in surrounding NA concentrations as occurs during sustained wakefulness and sleep periods. While speculative, activation of β2-AR fits our proposed model that includes a signal that accumulates during prolonged waking (e.g., NA) and triggers secondary events that have a time course similar to the discharge of NREM SWA; an entire process that occurs over hours in mammals [8,9]. Additional support for this idea is the observation that DSP-4 NA depletion specifically reduces energy in the slower delta bands of NREMS (<2.0 Hz) [15]. Similar findings are reported after inducible reductions in astroglial gliotransmission [2], intracellular Ca^2+^ [3], and β2-AR (present findings).

### 3.3. Astroglial β2-AR Signaling and Sleep Homeostasis

Our findings are consistent with ‘energy-charge’-based theories that link changes in astroglial energy substrates with sleep drive [40,41]. As proposed by Benington and Heller [42], waking activation of astroglial NA receptors triggers glycogenolysis and glucose release, which supports neuronal metabolic activity. The progressive depletion of this astroglial energy store during wakefulness increases NREM SWA via the release of neuronal adenosine [42]. The NA receptor subtype mediating glycogenolysis was not specified, but this likely involves the β2-AR [25,43]. While the original glycogen theory of sleep homeostasis has received mixed experimental support [40,41], activation of astroglial β2-ARs also increases the transfer of another energy source (lactate) to neurons via the astrocyte–neuron lactate shuttle (ANLS) [44]. There are several suggestive findings linking the ANLS and related metabolic processes to sleep homeostasis. Astroglial lactate mobilization increases during wakefulness and decreases during NREMS at a rate positively correlated with NREM SWA [45,46,47]. SDep also upregulates ANLS-related genes specifically in astrocytes [48], and disrupting lactate transport via KO of astroglial connexin 43 decreases wakefulness amounts and wakefulness bout duration during the active phase [49]. These results are similar to our findings of a longer siesta (Figure 1) and reduced sleep continuity post-SDep (Figure 2) in the cKO mice. Collectively, these data support the idea that β2-AR-mediated changes in astroglial energy substrates influence sleep expression and homeostasis.

### 3.4. Conclusions and Future Directions

We show that NA signaling in astroglial β2-AR may provide a link between wakefulness and subsequent sleep drive. This is consistent with our proposed model of sleep homeostasis comprised of three components: a neuronal signal that accumulates during wakefulness, an astroglial transducer/integrator of this signal, and a feedback mechanism that dampens the waking signal [8,9]. We previously identified putative components of a feedback mechanism (gliotransmission of ATP > adenosine [2]), an integrator of a waking signal (intracellular calcium [3]), and now the potential waking signal (NA) and its astroglial transducer (β2-AR). Nevertheless, there are many unanswered questions. First, the contribution of other astroglial NA receptor subtypes to sleep is unknown. While we think it likely that these are β-ARs (see above), similar studies using cKO of α-ARs are needed to answer this question. It is interesting, however, that cKO of astroglial β2-AR only partially reproduces changes in sleep homeostasis following brain depletion of NA in rats [15]. This suggests that other NA receptors may be involved. Second, while we discuss one possible connection between β2-AR (metabolism) and sleep homeostasis, other mechanisms are also possible. For example, activation of α-AR triggers gliotransmission of ATP which is hydrolyzed to the sleep-inducing molecule adenosine [50]. The role of β2-AR in gliotransmission is less understood, but if they have similar effects, then it is conceivable cKO of β2-AR would reduce gliotransmission of somnogens. Third, we do not know where in the brain astroglial β2-ARs exert their effects on sleep. The distribution of different subtypes of astroglial NA receptors in the mammalian brain is relatively unexplored, although there is evidence α-ARs and β-ARs are located in cortical and subcortical regions [25,38,51,52,53]. Therefore, as previously suggested [8,9], astrocytes via NA signaling could influence sleep via action in canonical subcortical sleep or wake centers, or through modulation of cortical activity. Fourth, further exploration of potential sex differences for these factors is needed because there is evidence for different noradrenergic neurocircuitry between male and female mice [54,55]. Lastly, more work is needed to determine if this signaling pathway is evolutionarily conserved. In mice and flies (*Drosophila melanogaster*), astroglial intracellular Ca^2+^ rises and falls with sleep drive and influences the homeostatic response to sleep loss [3,7,8]. Interestingly, sleep homeostasis in flies requires the astroglial expression of the monoaminergic receptor TyrRII [7]. Astroglial KO of TyrRII in flies reduces the homeostatic response to sleep loss in a manner similar to what we report in mice with cKO of astroglial β2-AR. Finally, β2-AR cKO mice unexpectedly have a longer ‘siesta’—a ~2 h sleep period that typically occurs during the latter half of the active phase. The timing of the siesta is thought to reflect the clock and homeostatic processes [56]. Given that there were no differences in baseline NREM SWA between cKO and WT mice, the longer siesta in cKO mice might be due to altered circadian processes that could involve astroglial activity in the suprachiasmatic nucleus—the primary pacemaker of circadian rhythms [57,58]. Astrocytes influence circadian rhythms [59], but the role of astroglial β2-ARs in central or peripheral clocks requires further investigation.

## 4. Materials and Methods

### 4.1. Animals

B6.Cg-Tg(GFAP-cre/ERT2)505Fmv/J (GFAP-CreERT2; #012849) mice were obtained from The Jackson Laboratory (Bar Harbor, ME, USA), and Adrb2^tm1Kry^ (Adrb2^flox^) mice on a C57Bl/6J background were obtained from Dr. Gerard Karsenty at Columbia University (New York, NY, USA) [60]. Breeding pairs of hemizygous GFAP-CreERT2^Tg+/−^ male mice and homozygous Adrb2^fl/fl^ female mice were established to obtain GFAP-Cre/ERT2^−/−^;Adrb2^fl/fl^ WT mice and GFAP-Cre/ERT2^Tg+/−^;Adrb2^fl/fl^ cKO mutant mice. We previously verified selective Cre recombination in astrocytes of GFAP-Cre/ERT2^Tg+/^^−^ mice [3], and we further verified astroglial β2-AR conditional knockdown using a functional Ca^2+^ imaging assay (see below). Mice were housed in standard cages on 24 ± 1 °C on a 12:12 h light:dark cycle with food and water ad libitum. All experimental procedures were approved by the Institutional Animal Care and Use Committee of Washington State University and conducted in accordance with National Research Council guidelines and regulations for experiments on live animals.

### 4.2. Surgical Procedures

#### 4.2.1. EEG and EMG Implantation

Adult male and female mice (WT (*n* = 13; females = 2) and cKO (*n* = 13; females = 2); 11–14-weeks old) were stereotaxically implanted with EEG and electromyographic (EMG) electrodes under isoflurane anesthesia according to previously published methods [3,61,62]. Briefly, four stainless steel screw electrodes (BC-002MPU188, Bellcan International Corp, Hialeah, FL, USA) were implanted contralaterally over frontal (2) and parietal (2) cortices, and 2 EMG wire electrodes were inserted in the nuchal muscles. Mice were allowed 5 days of recovery from surgery prior to habituation to the recording environment.

#### 4.2.2. Cranial Window for Ca^2+^ Imaging

Adult male and female mice (WT (*n* = 3; female = 1) and cKO (*n* = 3; female = 1); 14–18-weeks old) were anesthetized using isoflurane and placed in a stereotaxic frame for AAV2/5 *GfaABC_1_D*-GCaMP6f delivery and cranial window implantation as previously described [3]. A 3 mm craniotomy was made over the frontal cortex leaving the dura intact. AAV2/5 *GfaABC_1_D*-GCaMP6f (3.31 × 10^13^ GC/mL; Penn Vector Core, Philadelphia, PA, USA) was injected at two adjacent sites (1.5 µL each site; 200 nl/min) in the frontal cortex (AP: 2.0–2.5 mm, ML: −1.25–−1.75 mm, DV: −0.18 mm). The needle remained in place for 10 min after each injection. After vector delivery, a 3 mm glass coverslip was fixed over the craniotomy with cyanoacrylate adhesive and the skull covered with dental acrylic. After 2–4 weeks recovery, mice were fitted with a baseplate under isoflurane anesthesia. This recovery window allowed enough time to detect the fluorescent indicator [3,63,64]. The baseplate was secured to the skull with dental acrylic mixed with black carbon powder to house the miniature microscope (nVista 2.0; Inscopix, Palo Alto, CA, USA).

#### 4.2.3. Telemeter Implantation for Core Body Temperature

Adult male and female mice (WT (*n* = 2; female = 1) and cKO (*n* = 5; female = 1); 12–15-weeks old) were implanted with a telemetry device (G2 E-mitter, STARR Life Sciences Corp., Oakmont, PA, USA) in the peritoneal cavity under isoflurane anesthesia as previously described [3]. The telemeter was secured to the abdominal musculature with a suture. The skin was closed with wound clips which were removed after 8 days of recovery. During the recovery period, body weight, hydration, and fecal output were monitored daily.

### 4.3. Tamoxifen Injections

Prior to all surgical procedures, all mice were injected with tamoxifen (180 mg/kg; #T5648, Sigma-Aldrich, St. Louis, MO, USA) intraperitoneally once per day for 5 consecutive days alternating sides as previously described [3,65]. Tamoxifen was sonicated and dissolved in a solution of 90% sunflower oil (#S5007, Sigma-Aldrich) with 10% ethanol for a final concentration of 30 mg/mL. The tamoxifen solution was then sterile filtered through a 0.22 µm filter. Mice received 1 mL lactated Ringer’s solution with 5% dextrose subcutaneously daily until they started regaining body weight, which was monitored daily. Mice were given at least 10 days of recovery after the final tamoxifen injection prior to surgical procedures.

### 4.4. Experimental Procedures

#### 4.4.1. Sleep Phenotyping

Following postoperative recovery from EEG and EMG implantation, each mouse was placed in its own polycarbonate recording cage and connected to a lightweight, flexible recording cable. Mice acclimated to the recording cable and environment for at least 3 days prior to data collection. After acclimation, mice underwent a 24 h undisturbed baseline EEG/EMG recording starting at light onset. The next day, mice were sleep deprived for 6 h starting at light onset using the gentle handling technique as previously described [2,66]. Sleep deprivation by gentle handling involves introducing stimuli (e.g., tactile, auditory) to a mouse when the EEG/EMG and behavior (e.g., posture, quiescence) is indicative of sleep. Mice were then left undisturbed for the remaining 18 h (recovery period).

#### 4.4.2. Ca^2+^ Response to Isoproterenol

Ca^2+^ imaging started 2–5 weeks after surgical procedures to allow for postoperative recovery and fluorescent indicator expression [3,63,64]. On the experimental day, mice were attached to the miniature microscope and placed in the recording chamber. Mice were allowed at least 10 min to settle in the familiar chamber to which they were previously habituated. Next, baseline image capture occurred for 5 min. Mice were then injected intraperitoneally with 5 mg/kg of the β-AR agonist (-)-isoproterenol hydrochloride (#I6504, Sigma-Aldrich) made in saline [67,68]. Image capture occurred 20 min post-injection for 2 min. Mice were undisturbed during recordings.

#### 4.4.3. Daily Core Body Temperature Patterns

After postoperative recovery from abdominal transmitter implantation, mice were individually housed in standard mouse cages and allowed at least 5 days to habituate to the recording environment. After habituation, mice were recorded for 7 days under 12:12 h light:dark conditions. During this time, core body temperature was recorded continuously.

### 4.5. Data Acquisition and Processing

#### 4.5.1. EEG and EMG

EEG and EMG data were collected with a Grass 7 polygraph system (Natus Medical Incorporated, Pleasanton, CA, USA) via a lightweight recording cable for sleep phenotyping experiments. EEG and EMG signals were amplified and digitized at 256 Hz using Vital Recorder acquisition software (v3.0.0.0; SleepSign for Animal, Kissei Comtec Co., Ltd., Nagano, Japan). EEG and EMG data were high- and low-pass filtered at 0.3 and 100 Hz and 10 and 100 Hz, respectively [3]. 

EEG and EMG data were scored using SleepSign for Animal (v3.3.8.1803; Kissei Comtec Co., Ltd.) to assign vigilance states. Wakefulness, NREMS, and REMS (Figure S3) were scored by visual inspection of the EEG waveform, EMG activity, and fast Fourier transform (FFT) analysis using 4 s epochs by an experimenter blinded to genotype. Bout lengths were defined as ≥7 consecutive epochs (≥28 s) for wakefulness and NREMS and ≥4 consecutive epochs (≥16 s) for REMS [3]. Time spent in each state was expressed as a percentage of total recording time in 2 h bins. Frequency (number of bouts per 2 h) and duration of wakefulness, NREMS, and REMS bouts were expressed in 12 h bins for baseline conditions. Bout frequency and duration after SDep were expressed as differences from baseline by subtracting baseline values from SDep values [61,66,69]. These differences were then shown in 6 h and 12 h bins for the light and dark periods, respectively, for the post-SDep recovery phase.

FFT of the EEG was used to produce power spectra between 0–20 Hz with 0.5 Hz resolution. Delta (δ) was defined as 0.5–4 Hz and low delta as 0.5–1.5 Hz [2]. For genotypic comparisons of (1) baseline EEG spectral data and (2) NREMS EEG spectral data during the first 6 h of recovery sleep post-SDep, each spectral bin was expressed as a percentage of the total power in baseline wakefulness, NREMS, and REMS averaged across the three vigilance states. For hourly NREM delta power (i.e., NREM SWA) analysis after SDep, spectral values within the delta band or low delta band for each hour were normalized to the average NREM delta or low delta band value, respectively, from the last 4 h of the baseline light period (h9–12) and expressed as a percentage shown in 2 h bins [70]. EEG epochs with visually detected artifacts were excluded from spectral analyses.

#### 4.5.2. Ca^2+^ Imaging

Ca^2+^ imaging data was acquired through the head-mountable epifluorescent miniature microscope and Inscopix nVista HD software (v2.0.4) as previously described [3]. Imaging frames were captured at 10.1 frames per second with an exposure time of 49.664 ms at a gain of 2.0. LED power ranged from 50–80% to adjust the upper tail of the histogram to be as close to a pixel value of 1500 to ensure good signal-to-noise ratio. The LED power was set at the beginning of the experiment for each mouse and did not change for the remainder of the experiment.

Imaging data were preprocessed using the Data Processing Software (v1.2.1; Inscopix) as previously described [3]. For this preprocessing, movies were spatially and temporally downsampled by a factor of 2 and 5, respectively, to reduce the data footprint, and defective pixels were rectified [71]. Small lateral displacements were then corrected using the motion correction algorithm in the Data Processing Software. Next, regions of interest (ROIs) were selected by manually identifying cell-body-sized, high-contrast regions over the course of the recording, and contours were drawn to contain pixels from the ROI as described previously [3,72]. ROIs were further validated by inspecting temporal traces of each ROI for Ca^2+^ signals consistent with Ca^2+^ transients from individual cells [3,63,73,74,75]. We identified 115 ROIs from 3 WT mice and 84 ROIs from 3 cKO mice. Raw fluorescent values were exported for each ROI for the entire recording. To correct for slight decays in fluorescent signal across each recording, Ca^2+^ traces for each ROI were detrended by subtracting an exponential curve fit from each individual raw Ca^2+^ imaging trace using the ‘fit’ function from MATLAB’s (R2019a; MathWorks, Natick, MA, USA) Curve Fitting toolbox [76] as previously described [3]. The curve fit was added back to each trace to bring traces to a common baseline. Ca^2+^ values were then expressed as percent change from the median fluorescent value of the entire recording for each ROI [3,76,77]. Event detection with a 0.5% prominence threshold was then performed using the ‘findpeaks’ function from MATLAB’s Signal Processing toolbox as previously described [3]. Two conditions had to be met to identify an ROI’s frame as an event: (1) the value of that frame is larger than its two neighboring frames, and (2) the difference between that value and the value of the larger neighboring trough (prominence) is greater than 0.5% of the range of the entire trace. Identified events were then used to determine frequency of Ca^2+^ events per 5 s.

#### 4.5.3. Core Body Temperature

Core body temperature was recorded using VitalView Activity Data Acquisition software (v5.1; STARR Life Sciences Corp.) with 10 min resolution. Data were calculated in 2 h bins to assess diurnal/nocturnal patterns and as 7 d means of minimum and maximum values to determine if temperatures were within normal range. 

### 4.6. Statistical Analysis

Plots were generated in SigmaPlot (v11.0, Systat Software, Inc., San Jose, CA, USA) and R (v4.1.1), and statistical analyses were performed using SPSS for Windows 25 (IBM Corporation, Armonk, NY, USA). Data are presented as means ± standard error of the mean (SE) unless otherwise stated. Normality of the data was determined with Shapiro–Wilk or Kolmogorov–Smirnov tests. A general linear model for repeated measures (RM) using time (hours) as the repeated measure and genotype (WT vs. cKO) as the between-subjects factor was used when multiple measurements were made over time (i.e., time-in-state, bout frequency, bout duration, NREM delta power, core body temperature). Baseline time-in-state RM comparisons were made over all time intervals during the light (h1–12) and dark (h13–24) periods. For recovery data post-SDep, RM comparisons were made over all time intervals during the light (h7–12) and dark (h13–24) recovery periods for time-in-state and NREM delta power data. Bout data RM comparisons were made over all time intervals during the full 24 h recording period (h1–24) for baseline and SDep recovery. RM was also used to compare normalized EEG spectral power using frequency (Hz) as the repeated measure from 0–20 Hz, spectral power as the dependent variable, and genotype (WT vs. cKO) as the between-subjects factor. RM comparisons were tested for sphericity, and a Greenhouse–Geisser correction was applied when appropriate. Post-hoc pairwise comparisons using Sidak corrections were performed when there were significant interaction effects or main effects of genotype. Baseline NREM delta power was assessed with a Kruskal–Wallis test due to normal periods of prolonged wakefulness during the dark (i.e., active) period. Genotypic comparisons of Ca^2+^ event frequency and ΔF/F values under baseline conditions and in response to isoproterenol were made using a Mann–Whitney U test. For simplicity, outliers were not plotted with the boxplots. Body mass at the time of surgery (post-tamoxifen treatment) was compared using a Mann–Whitney U test. Unpaired Student’s t-tests with genotype as the grouping variable were used for 7 d minimum and maximum core body temperature comparisons. An alpha level less than 0.05 was used to indicate significance.

## Data Availability

Data available upon request.

## Supplementary Materials

The following supporting information can be downloaded at: https://www.mdpi.com/article/10.3390/clockssleep4030028/s1, Figure S1: Verification of Astroglial β2-AR cKO, Figure S2: Assessment of Astroglial β2-AR cKO on Electroencephalographic Activity and Physiology, Figure S3: Arousal State-specific Electroencephalography (EEG) and Electromyography (EMG) from a WT Mouse and a cKO Mouse.

## References

[1] Dijk D.J., Lockley S.W. (2002). Integration of human sleep-wake regulation and circadian rhythmicity. J. Appl. Physiol..

[2] Halassa M.M., Florian C., Fellin T., Munoz J.R., Lee S.Y., Abel T., Haydon P.G., Frank M.G. (2009). Astrocytic modulation of sleep homeostasis and cognitive consequences of sleep loss. Neuron.

[3] Ingiosi A.M., Hayworth C.R., Harvey D.O., Singletary K.G., Rempe M.J., Wisor J.P., Frank M.G. (2020). A role for astroglial calcium in mammalian sleep and sleep regulation. Curr. Biol..

[4] Bojarskaite L., Bjørnstad D.M., Pettersen K.H., Cunen C., Hermansen G.H., Åbjørsbråten K.S., Chambers A.R., Sprengel R., Vervaeke K., Tang W. (2020). Astrocytic Ca^2+^ signaling is reduced during sleep and is involved in the regulation of slow wave sleep. Nat. Commun..

[5] Vaidyanathan T.V., Collard M., Yokoyama S., Reitman M.E., Poskanzer K.E. (2021). Cortical astrocytes independently regulate sleep depth and duration via separate gpcr pathways. eLife.

[6] Tsunematsu T., Sakata S., Sanagi T., Tanaka K.F., Matsui K. (2021). Region-specific and state-dependent astrocyte Ca^2+^ dynamics during the sleep-wake cycle in mice. J. Neurosci..

[7] Blum I.D., Keles M.F., Baz E.S., Han E., Park K., Luu S., Issa H., Brown M., Ho M.C.W., Tabuchi M. (2021). Astroglial calcium signaling encodes sleep need in drosophila. Curr. Biol..

[8] Ingiosi A.M., Frank M.G. Goodnight, astrocyte: Waking up to astroglial mechanisms in sleep. FEBS J..

[9] Frank M.G. (2013). Astroglial regulation of sleep homeostasis. Curr. Opin. Neurobiol..

[10] Jones B.E. (1991). The role of noradrenergic locus coeruleus neurons and neighboring cholinergic neurons of the pontomesencephalic tegmentum in sleep-wake states. Progress. Brain Res..

[11] Jones B.E., Kryger M.H., Roth T., Dement W.C. (2005). Basic mechanisms of sleep-waking states. Principles and Practice of Sleep Medicine.

[12] Daniele T.M.d.C., de Bruin P.F.C., Rios E.R.V., de Bruin V.M.S. (2017). Effects of exercise on depressive behavior and striatal levels of norepinephrine, serotonin and their metabolites in sleep-deprived mice. Behav. Brain Res..

[13] Bellesi M., Tononi G., Cirelli C., Serra P.A. (2016). Region-specific dissociation between cortical noradrenaline levels and the sleep/wake cycle. Sleep.

[14] Bekar L.K., He W., Nedergaard M. (2008). Locus coeruleus alpha-adrenergic-mediated activation of cortical astrocytes in vivo. Cereb. Cortex.

[15] Cirelli C., Huber R., Gopalakrishnan A., Southard T.L., Tononi G. (2005). Locus ceruleus control of slow-wave homeostasis. J. Neurosci..

[16] Ding F., O’Donnell J., Thrane A.S., Zeppenfeld D., Kang H., Xie L., Wang F., Nedergaard M. (2013). A1-adrenergic receptors mediate coordinated Ca^2+^ signaling of cortical astrocytes in awake, behaving mice. Cell Calcium.

[17] Chow L.M.L., Zhang J., Baker S. (2008). Inducible cre recombinase activity in mouse mature astrocytes and adult neural precursor cells. Trangenic Res..

[18] Hanada R., Leibbrandt A., Hanada T., Kitaoka S., Furuyashiki T., Fujihara H., Trichereau J., Paolino M., Qadri F., Plehm R. (2009). Central control of fever and female body temperature by rankl/rank. Nature.

[19] Ganat Y.M., Silbereis J., Cave C., Ngu H., Anderson G.M., Ohkubo Y., Ment L.R., Vaccarino F.M. (2006). Early postnatal astroglial cells produce multilineage precursors and neural stem cells in vivo. J. Neurosci..

[20] Kim J.G., Suyama S., Koch M., Jin S., Argente-Arizon P., Argente J., Liu Z.W., Zimmer M.R., Jeong J.K., Szigeti-Buck K. (2014). Leptin signaling in astrocytes regulates hypothalamic neuronal circuits and feeding. Nat. Neurosci..

[21] García-Cáceres C., Quarta C., Varela L., Gao Y., Gruber T., Legutko B., Jastroch M., Johansson P., Ninkovic J., Yi C.X. (2016). Astrocytic insulin signaling couples brain glucose uptake with nutrient availability. Cell.

[22] Franco C., Genis L., Navarro J.A., Perez-Domper P., Fernandez A.M., Schneuwly S., Torres Alemán I. (2017). A role for astrocytes in cerebellar deficits in frataxin deficiency: Protection by insulin-like growth factor i. Mol. Cell. Neurosci..

[23] Koeppen J., Nguyen A.Q., Nikolakopoulou A.M., Garcia M., Hanna S., Woodruff S., Figueroa Z., Obenaus A., Ethell I.M. (2018). Functional consequences of synapse remodeling following astrocyte-specific regulation of ephrin-b1 in the adult hippocampus. J. Neurosci..

[24] Jin S., Kim K.K., Park B.S., Kim D.H., Jeong B., Kang D., Lee T.H., Park J.W., Kim J.G., Lee B.J. (2020). Function of astrocyte myd88 in high-fat-diet-induced hypothalamic inflammation. J. Neuroinflam..

[25] Gao V., Suzuki A., Magistretti P.J., Lengacher S., Pollonini G., Steinman M.Q., Alberini C.M. (2016). Astrocytic β_2_-adrenergic receptors mediate hippocampal long-term memory consolidation. Proc. Natl. Acad. Sci. USA.

[26] Hofstetter J.R., Svihla-Jones D.A., Mayeda A.R. (2007). A QTL on mouse chromosome 12 for the genetic variance in free-running circadian period between inbred strains of mice. J. Circadian Rhythm..

[27] Franken P., Malafosse A., Tafti M. (1999). Genetic determinants of sleep regulation in inbred mice. Sleep.

[28] Franken P., Lopez-Molina L., Marcacci L., Schibler U., Tafti M. (2000). The transcription factor dbp affects circadian sleep consolidation and rhythmic eeg activity. J. Neurosci..

[29] Jouvet M. (1999). Sleep and serotonin: An unfinished story. Neuropsychopharmacology.

[30] Greene R.W., Frank M.G. (2010). Slow wave activity during sleep: Functional and therapeutic implications. Neuroscientist.

[31] Sallanon M., Janin M., Buda C., Jouvet M. (1983). Serotonergic mechanisms and sleep rebound. Brain Res..

[32] Tobler I., Borbely A.A. (1982). Sleep regulation after reduction of brain serotonin: Effect of p-chlorophenylalanine combined with sleep deprivation in the rat. Sleep.

[33] Gerstner J.R., Koberstein J.N., Watson A.J., Zapero N., Risso D., Speed T.P., Frank M.G., Peixoto L. (2016). Removal of unwanted variation reveals novel patterns of gene expression linked to sleep homeostasis in murine cortex. BMC Genom..

[34] Ouyang M., Hellman K., Abel T., Thomas S.A. (2004). Adrenergic signaling plays a critical role in the maintenance of waking and in the regulation of rem sleep. J. Neurophysiol..

[35] Mallick B.N., Alam M.N. (1992). Different types of norepinephrinergic receptors are involved in preoptic area mediated independent modulation of sleep-wakefulness and body temperature. Brain Res..

[36] Catus S.L., Gibbs M.E., Sato M., Summers R.J., Hutchinson D.S. (2011). Role of β-adrenoceptors in glucose uptake in astrocytes using β-adrenoceptor knockout mice. Br. J. Pharmacol..

[37] Hertz L., Lovatt D., Goldman S.A., Nedergaard M. (2010). Adrenoceptors in brain: Cellular gene expression and effects on astrocytic metabolism and [Ca(^2+^)]i. Neurochem. Int..

[38] Oe Y., Wang X., Patriarchi T., Konno A., Ozawa K., Yahagi K., Hirai H., Tsuboi T., Kitaguchi T., Tian L. (2020). Distinct temporal integration of noradrenaline signaling by astrocytic second messengers during vigilance. Nat. Commun..

[39] Wahis J., Holt M.G. (2021). Astrocytes, noradrenaline, α1-adrenoreceptors, and neuromodulation: Evidence and unanswered questions. Front. Cell. Neurosci..

[40] Scharf M.T., Naidoo N., Zimmerman J.E., Pack A.I. (2008). The energy hypothesis of sleep revisited. Prog. Neurobiol..

[41] Petit J.-M., Burlet-Godinot S., Magistretti P.J., Allaman I. (2015). Glycogen metabolism and the homeostatic regulation of sleep. Metab. Brain Dis..

[42] Benington J., Heller H.C. (1995). Restoration of brain energy metabolism as the function of sleep. Prog. Neurobiol..

[43] Dong J.-H., Chen X., Cui M., Yu X., Pang Q., Sun J.-P. (2012). Beta2-adrenergic receptor and astrocyte glucose metabolism. J. Mol. Neurosci..

[44] Pellerin L., Magistretti P.J. (2012). Sweet sixteen for anls. J. Cereb. Blood Flow Metab..

[45] Wisor J.P., Rempe M.J., Schmidt M.A., Moore M.E., Clegern W.C. (2013). Sleep slow-wave activity regulates cerebral glycolytic metabolism. Cereb. Cortex.

[46] Dash M.B., Tononi G., Cirelli C. (2012). Extracellular levels of lactate, but not oxygen, reflect sleep homeostasis in the rat cerebral cortex. Sleep.

[47] Naylor E., Aillon D.V., Barrett B.S., Wilson G.S., Johnson D.A., Johnson D.A., Harmon H.P., Gabbert S., Petillo P.A. (2012). Lactate as a biomarker for sleep. Sleep.

[48] Petit J.M., Gyger J., Burlet-Godinot S., Fiumelli H., Martin J.L., Magistretti P.J. (2013). Genes involved in the astrocyte-neuron lactate shuttle (anls) are specifically regulated in cortical astrocytes following sleep deprivation in mice. Sleep.

[49] Clasadonte J., Scemes E., Wang Z., Boison D., Haydon P.G. (2017). Connexin 43-mediated astroglial metabolic networks contribute to the regulation of the sleep-wake cycle. Neuron.

[50] Bazargani N., Attwell D. (2017). Amines, astrocytes, and arousal. Neuron.

[51] Cash R., Raisman R., Lanfumey L., Ploska A., Agid Y. (1986). Cellular localization of adrenergic receptors in rat and human brain. Brain Res..

[52] Zhang Y., Chen K., Sloan S.A., Bennett M.L., Scholze A.R., O’Keeffe S., Phatnani H.P., Guarnieri P., Caneda C., Ruderisch N. (2014). An rna-sequencing transcriptome and splicing database of glia, neurons, and vascular cells of the cerebral cortex. J. Neurosci..

[53] Ramos B.P., Arnsten A.F. (2007). Adrenergic pharmacology and cognition: Focus on the prefrontal cortex. Pharmacol. Ther..

[54] Mulvey B., Bhatti D.L., Gyawali S., Lake A.M., Kriaucionis S., Ford C.P., Bruchas M.R., Heintz N., Dougherty J.D. (2018). Molecular and functional sex differences of noradrenergic neurons in the mouse locus coeruleus. Cell Rep..

[55] Sun P., Wang J., Zhang M., Duan X., Wei Y., Xu F., Ma Y., Zhang Y.-H. (2020). Sex-related differential whole-brain input atlas of locus coeruleus noradrenaline neurons. Front. Neural Circuits.

[56] Ehlen J.C., Jones K.A., Pinckney L., Gray C.L., Burette S., Weinberg R.J., Evans J.A., Brager A.J., Zylka M.J., Paul K.N. (2015). Maternal ube3a loss disrupts sleep homeostasis but leaves circadian rhythmicity largely intact. J. Neurosci..

[57] Brancaccio M., Patton A.P., Chesham J.E., Maywood E.S., Hastings M.H. (2017). Astrocytes control circadian timekeeping in the suprachiasmatic nucleus via glutamatergic signaling. Neuron.

[58] Brancaccio M., Edwards M.D., Patton A.P., Smyllie N.J., Chesham J.E., Maywood E.S., Hastings M.H. (2019). Cell-autonomous clock of astrocytes drives circadian behavior in mammals. Science.

[59] Astiz M., Delgado-García L.M., López-Mascaraque L. (2021). Astrocytes as essential time-keepers of the central pacemaker. Glia.

[60] Hinoi E., Gao N., Jung D.Y., Yadav V., Yoshizawa T., Myers M.G., Chua S.C., Kim J.K., Kaestner K.H., Karsenty G. (2008). The sympathetic tone mediates leptin’s inhibition of insulin secretion by modulating osteocalcin bioactivity. J. Cell Biol..

[61] Ingiosi A.M., Schoch H., Wintler T., Singletary K.G., Righelli D., Roser L.G., Medina E., Risso D., Frank M.G., Peixoto L. (2019). Shank3 modulates sleep and expression of circadian transcription factors. eLife.

[62] Frank M.G., Stryker M.P., Tecott L.H. (2002). Sleep and sleep homeostasis in mice lacking the 5-ht2c receptor. Neuropsychopharmacology.

[63] Srinivasan R., Huang B.S., Venugopal S., Johnston A.D., Chai H., Zeng H., Golshani P., Khakh B.S. (2015). Ca^2+^ signaling in astrocytes from ip3r2−/− mice in brain slices and during startle responses in vivo. Nat. Neurosci..

[64] Heuser K., Nome C.G., Pettersen K.H., Åbjørsbråten K.S., Jensen V., Tang W., Sprengel R., Taubøll E., Nagelhus E.A., Enger R. (2018). Ca^2+^ signals in astrocytes facilitate spread of epileptiform activity. Cereb. Cortex.

[65] Bjorness T.E., Dale N., Mettlach G., Sonneborn A., Sahin B., Fienberg A.A., Yanagisawa M., Bibb J.A., Greene R.W. (2016). An adenosine-mediated glial-neuronal circuit for homeostatic sleep. J. Neurosci. Off. J. Soc. Neurosci..

[66] Ingiosi A.M., Raymond R.M., Pavlova M.N., Opp M.R. (2015). Selective contributions of neuronal and astroglial interleukin-1 receptor 1 to the regulation of sleep. Brain. Behav. Immun..

[67] Gonzalez J.P., Ramachandran J., Xie L.H., Contreras J.E., Fraidenraich D. (2015). Selective connexin43 inhibition prevents isoproterenol-induced arrhythmias and lethality in muscular dystrophy mice. Sci. Rep..

[68] Ji S., Guo R., Wang J., Qian L., Liu M., Xu H., Zhang J., Guan Y., Yang G., Chen L. (2020). Mpges-1 deletion attenuates isoproterenol-induced myocardial fibrosis in mice. J. Pharmacol. Exp. Ther..

[69] Ingiosi A.M., Opp M.R. (2016). Sleep and immunomodulatory responses to systemic lipopolysaccharide in mice selectively expressing interleukin-1 receptor 1 on neurons or astrocytes. Glia.

[70] Franken P., Tobler I., Borbely A.A. (1991). Sleep homeostasis in the rat: Simulation of the time course of eeg slow-wave activity. Neurosci. Lett..

[71] Gulati S., Cao V.Y., Otte S. (2017). Multi-layer cortical Ca^2+^ imaging in freely moving mice with prism probes and miniaturized fluorescence microscopy. J. Vis. Exp..

[72] Weber F., Hoang Do J.P., Chung S., Beier K.T., Bikov M., Saffari Doost M., Dan Y. (2018). Regulation of rem and non-rem sleep by periaqueductal gabaergic neurons. Nat. Commun..

[73] Jimenez J.C., Su K., Goldberg A.R., Luna V.M., Biane J.S., Ordek G., Zhou P., Ong S.K., Wright M.A., Zweifel L. (2018). Anxiety cells in a hippocampal-hypothalamic circuit. Neuron.

[74] Kirschen G.W., Shen J., Tian M., Schroeder B., Wang J., Man G., Wu S., Ge S. (2017). Active dentate granule cells encode experience to promote the addition of adult-born hippocampal neurons. J. Neurosci..

[75] Resendez S.L., Jennings J.H., Ung R.L., Namboodiri V.M., Zhou Z.C., Otis J.M., Nomura H., McHenry J.A., Kosyk O., Stuber G.D. (2016). Visualization of cortical, subcortical and deep brain neural circuit dynamics during naturalistic mammalian behavior with head-mounted microscopes and chronically implanted lenses. Nat. Protoc..

[76] Eban-Rothschild A., Rothschild G., Giardino W.J., Jones J.R., de Lecea L. (2016). Vta dopaminergic neurons regulate ethologically relevant sleep-wake behaviors. Nat. Neurosci..

[77] Paukert M., Agarwal A., Cha J., Doze V.A., Kang J.U., Bergles D.E. (2014). Norepinephrine controls astroglial responsiveness to local circuit activity. Neuron.

