# Supplementary material for: Noradrenergic Signaling in Astrocytes Influences Mammalian Sleep Homeostasis
Source: Clocks Sleep. 2022 Jul 7;4(3):332–45. doi: 10.3390/clockssleep4030028 (PMC9326550; doi:10.3390/clockssleep4030028)
Supplement: Supplementary file 1 [file clockssleep-04-00028-s001.zip › clockssleep-1761726-supplementary.pdf]

Supplementary Figures

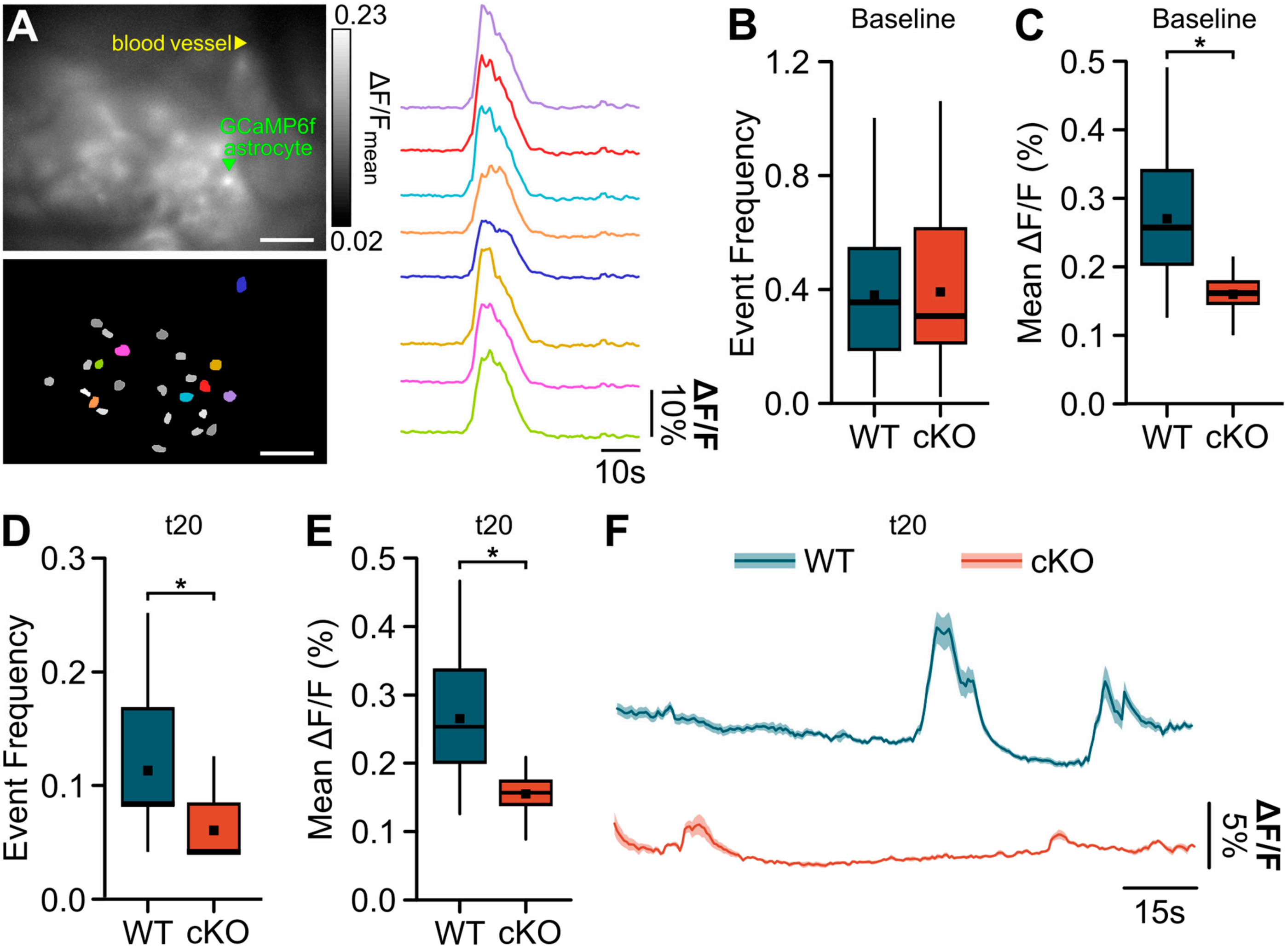

Figure S1. Verification of astroglial β2-AR cKO. (**A**) Top left panel is a representative maximum projection image from the miniature microscope. The yellow triangle points to a blood vessel, and the green triangle shows an example of a GCaMP6f$^+$ astrocyte. Bottom left panel shows locations of identified GCaMP6f$^+$ astrocytes/ROIs that correspond with the top left projection image. Right panel shows fluorescent GCaMP6f Ca^{2+} traces that correspond to the color-coded ROIs in the bottom left spatial map. Scale bars = 100 µm. Distribution of (**B**) frequency of events (event # per 5 s) and (**C**) mean ΔF/F values under baseline conditions (Mann-Whitney U). Black squares denote means. Note that the event frequency does not differ between WT and cKO mice despite overall lower mean ΔF/F values for cKO. Values are from n = 115 individual ROIs from 3 WT mice and n = 84 individual ROIs from 3 cKO mice. Distribution of (**D**) frequency of events (event # per 5 s) and (**E**) mean ΔF/F values at 20 min (t20) following intraperitoneal (i.p.) injection of the β-AR agonist isoproterenol (5 mg/kg) (Mann-Whitney U). Black squares denote means. Note that both event frequency and mean ΔF/F values are blunted for cKO mice in response to the β-AR agonist compared to WT mice. Values are from n = 115 individual ROIs from 3 WT mice and n = 84 individual ROIs from 3 cKO mice. (**F**) Representative Ca^{2+} traces (ΔF/F) from a WT mouse (23 ROIs) and a cKO mouse (23 ROIs) at 20 min (t20) following injection of isoproterenol (5 mg/kg, i.p.). Values are means ± standard deviation. * $p < 0.05$.

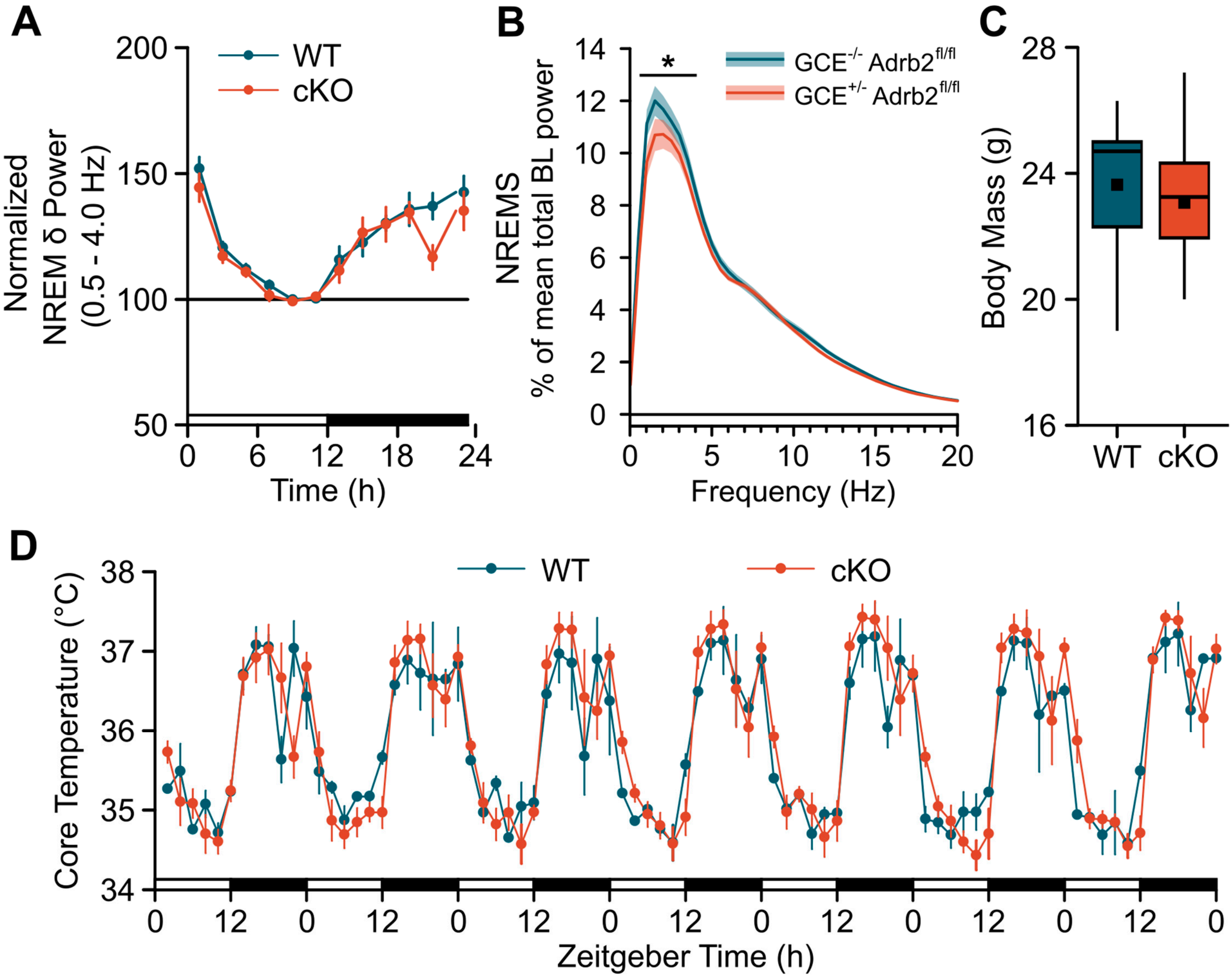

Figure S2. Assessment of astroglial β2-AR cKO on electroencephalographic activity and physiology. (**A**) Baseline normalized NREM SWA (i.e., delta (δ) power) shown in 2 h bins (Kruskal-Wallis). Open and closed bars on the x-axis denote the light and dark periods, respectively. Values are means ± SE. (**B**) Normalized EEG spectral power for NREMS during the first 6 h of recovery sleep post-sleep deprivation (ANOVA over delta (0.5 – 4 Hz; $F(1,205.87) = 9.07$, $p = 0.003$) and upper (4.5 – 20 Hz) bands). Values are means ± SE. (**C**) Body mass distribution of WT and cKO mice at time of surgery (Mann-Whitney U). Black squares denote means. (**D**) Continuous core body temperature measurements shown across 7 days in 2 h bins for WT and cKO mice (repeated measures ANOVA). Open and closed bars on the x-axis denote the light and dark periods, respectively. Values are means ± SE. n = 13 WT and n = 13 cKO mice for A – C; n = 2 WT and n = 5 cKO mice for D.

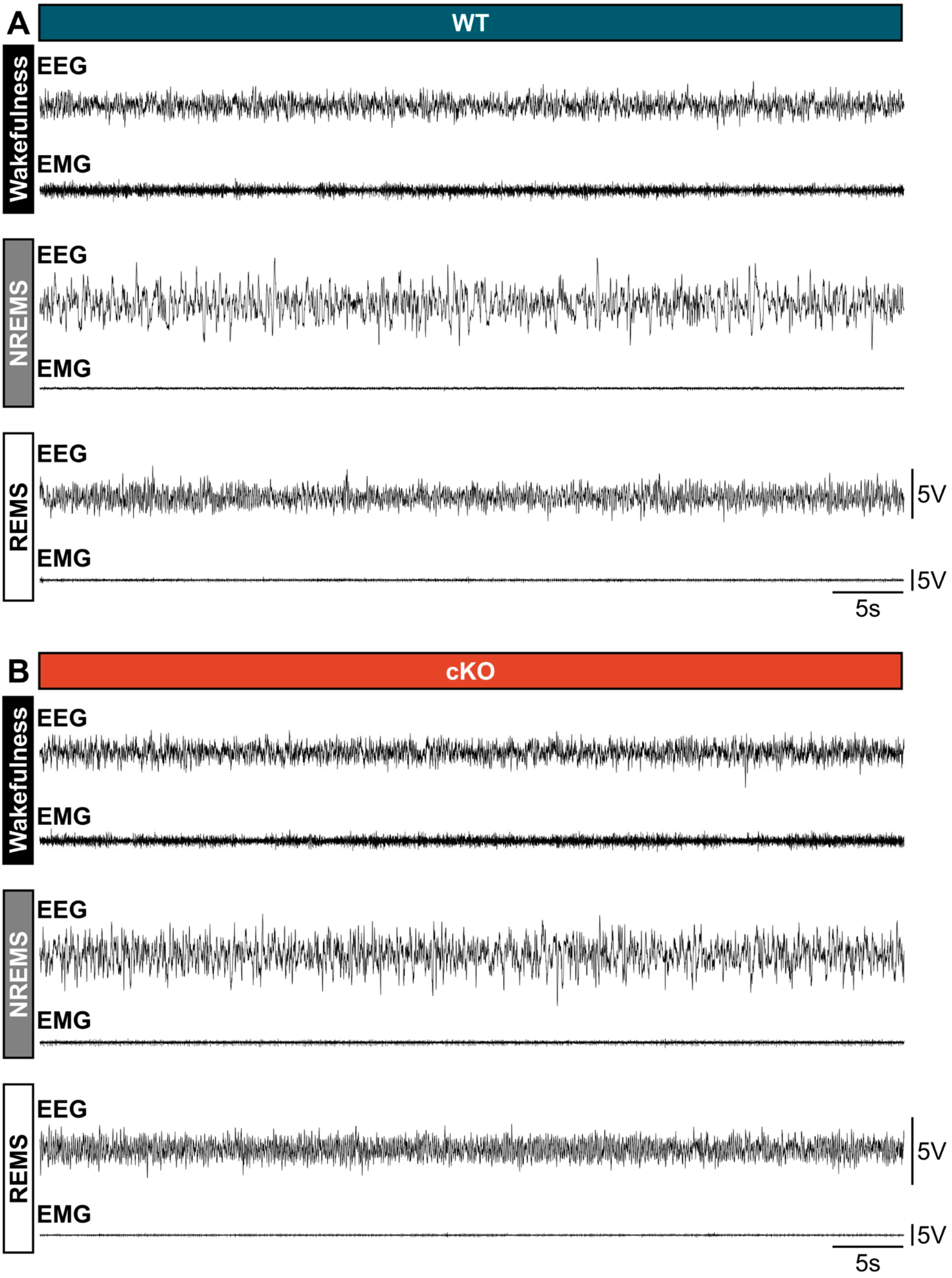

Figure S3. Arousal state-specific electroencephalography (EEG) and electromyography (EMG) from a WT mouse and a cKO mouse. Representative baseline EEG and EMG traces for a (**A**) WT mouse and a (**B**) cKO mouse shown for wakefulness (top), NREMS (middle), and REMS (bottom) for each mouse.
